# Editorial Change

**Published:** 1854-03

**Authors:** 


### EDITORIAL CHANGE.

Dr. JOSEPH PARRISH, who for a number of years has edited the "New Jersey Medical Reporter" with excellent taste and judgment, and given it a high character among the Medical Journals of our country, has gone to Philadelphia to reside, and the Reporter is to be edited, in future, by Dr. G. M. BUTLER.

